# Uterine rupture in pregnancy over 5 years: A retrospective descriptive study

**DOI:** 10.1177/17455057251399891

**Published:** 2025-11-21

**Authors:** António De Pinho, Francisco Martins Dos Santos, Olímpia Carmo, João Bernardes, Ana Reynolds

**Affiliations:** 1 Department of Gynaecology-Obstetrics and Paediatrics, Faculty of Medicine, University of Porto, Portugal; 2 Department of Gynaecology-Obstetrics, Local Health Unit of Tâmega and Sousa, Penafiel, Portugal; 3 Faculty of Medicine, University of Porto, Portugal; 4 CINTESIS@RISE, Faculty of Medicine, University of Porto, Portugal

**Keywords:** caesarean section, clinical presentation, labour, maternal morbidity, pregnancy, uterine rupture

## Abstract

**Background:**

Uterine rupture is a rare but serious complication in obstetrics, associated with maternal and fetal risks. This study evaluates its prevalence and outcomes in a Portuguese hospital.

**Objectives:**

To assess the prevalence of uterine rupture in pregnancy, as well as the associated maternal and neonatal morbidity and mortality, in a hospital setting in Portugal.

**Design:**

A retrospective descriptive study was conducted.

**Methods:**

Retrospective review of complete uterine rupture cases (2019–2024) at the Local Health Unit of Tâmega e Sousa. Data included maternal characteristics, obstetric history and outcomes.

**Results:**

Among 10,447 deliveries, 13 cases of uterine rupture occurred (12.44/10,000), primarily in women with prior caesarean sections (84.6%). Abnormal cardiotocography (76.9%) and abdominal pain (23.1%) were common signs. Fetal extrusion occurred in 69.2%. No maternal deaths were recorded, but perinatal mortality was 7.7%. Post-partum haemorrhage affected 61.5%, with five transfusions required.

**Conclusion:**

The prevalence of uterine rupture aligns with rates in developed countries. Caution is advised when using oxytocin in women with prior caesareans. Prompt detection and intervention are crucial to minimize complications.

## Introduction

Uterine rupture is a rare obstetric complication associated with elevated rates of maternal and fetal morbidity and mortality.^1^ It is defined as a clinically apparent complete separation of all layers of the uterus, occurring during or before labour.^2^ The initial signs and symptoms of uterine rupture are typically nonspecific, including fetal heart rate abnormalities, abdominal pain, and vaginal bleeding, making diagnosis challenging and potentially delaying definitive therapy. Consequently, a high index of diagnostic suspicion is required for timely recognition and intervention.^3,4^ Prompt surgical management of uterine rupture is critical, as it significantly reduces the risk of permanent fetal and maternal morbidity.^5^

Trial of labour after caesarean section (TOLAC) is a recognized risk factor for uterine rupture. Other associated risk factors include induction of labour, labour augmentation with oxytocin, fetal macrosomia, congenital uterine anomalies, prior myometrial surgery and short intervals between deliveries.^6–10^

The prevalence of uterine rupture in developed countries ranges from 0.5 to 7.9 per 10,000 births.^11^ Over recent decades, the incidence has increased, likely due to the growing number of patients undergoing TOLAC,^12^ and the rising global rates of caesarean deliveries.^13^

There is a scarcity of published data related to uterine rupture in Portugal. The latest report by the Portuguese Health Regulatory Authority (Entidade Reguladora da Saúde) documented a national caesarean section rate of 38%.^14^ In comparison, a 2021 report by the World Health Organization indicated a global average caesarean section rate of 21%,^15^ highlighting that Portugal’s caesarean rate seems significantly higher than the international average.

The primary objective of this study is to assess the prevalence of uterine rupture in pregnancy, as well as the associated maternal and neonatal morbidity and mortality, in a hospital setting in Portugal.

## Materials and methods

This study is a retrospective analysis including patients with uterine rupture managed at the Local Health Unit of Tâmega e Sousa, Penafiel, Porto, Portugal, between 2019 and 2024 (5 years). The hospital serves a population of approximately 500,000 individuals, with more than 2000 deliveries per year.^16^

The research received approval as an outcome analysis and was registered with the hospital’s Ethics Committee (number 33/2022). Informed consent was waived by the IRB committee due to the observational nature of the study and the use of de-identified data.

This study included all women with a confirmed diagnosis of complete uterine rupture managed at our institution between 2019 and 2024. Complete uterine rupture was defined as a full-thickness separation of all layers of the uterine wall (including the serosa). Cases of uterine scar dehiscence – defined as partial disruption of the uterine wall without serosal breach – were excluded. Inclusion and exclusion decisions were based on intraoperative findings documented in surgical and clinical records.

The information was sourced from two distinct systems: a diagnosis source approach through *ObsCare*^®^, a specialized obstetric clinical records system and post-event hospital codification. A list of cases was generated based on the *International Statistical Classification of Diseases and Related Health Problems, 10th Revision* (ICD-10) codes: O71.0 (spontaneous rupture of the uterus before the onset of labour) and O71.1 (rupture of the uterus during labour). Maternal characteristics, obstetric history, antenatal and intrapartum events, along with maternal and neonatal outcomes were reviewed using electronic medical records.

All maternal and neonatal data were complete. There were no missing values for the variables analysed, and therefore no imputation or data exclusion was required.

No formal sample size calculation was performed prior to the study, as this was a retrospective analysis including all confirmed cases of complete uterine rupture during the study period. The total sample size was therefore determined by the number of eligible cases identified through hospital records between 2019 and 2024.

Data analysis was conducted using *SPSS*^®^ statistical software version 29.0.2.0 (20) (IBM Corp., Armonk, NY, USA).

The reporting of this study conforms to the Strengthening the Reporting of Observational Studies in Epidemiology (STROBE) guidelines for observational studies.^17^

## Results

During the 5-year study period, there were a total of 10,447 deliveries, of which 13 involved a complete uterine rupture, resulting in a prevalence of 12.44 per 10,000 deliveries. During this timeframe, the caesarean section rate at the institution was 25.99%. A total of 550 women underwent induction of labour following a previous caesarean section.

All cases of uterine rupture occurred in the third trimester and were associated with labour, labour induction or elective caesarean section. The mean gestational age at the time of rupture was 38.46 weeks (Supplemental Table S1). No cases of uterine rupture during pregnancy outside the context of labour or surgical delivery were observed. The mean body mass index was 27.77 kg/m^2^. Among the 13 women who experienced complete uterine rupture, 11 (84.6%) had a scarred uterus due to a previous caesarean section, with a mean inter-pregnancy interval of 94.64 months following their surgery. No cases were reported in women with a history of other uterine surgeries, such as myomectomy. The majority of these women underwent spontaneous labour, with two cases (15.4%) involving induction of labour after a previous caesarean. The prevalence of uterine rupture in inductions after caesarean was 36.4 per 10,000 deliveries. In both cases, labour induction was performed using oxytocin infusion. Additionally, in 10 out of the 13 cases, oxytocin was utilized for labour augmentation.

The most common sign of uterine rupture (Supplemental Table S2) was an abnormal cardiotocography (CTG), observed in 10 women (76.9%). Other signs/symptoms were much less frequent, with abdominal pain being the second most common, reported by three women (23.1%). Vaginal bleeding, shoulder pain and loss of fetal station were also represented. Notably, two women (15.4%) were asymptomatic, with the diagnosis of uterine rupture made intraoperatively during caesarean section (one elective and another due to prolonged labour).

Of the 13 patients with complete uterine rupture, there were no maternal deaths nor peripartum hysterectomies (Supplemental Table S3). The most common complication was fetal extrusion from the uterus, occurring in nine cases (69.2%). The majority of ruptures occurred at the lower uterine segment, from extension to the previous caesarean scar, one of them with extension to the cervix and another to the bladder. In the two cases of rupture without previous uterine scar, both were also located in the lower uterine segment. One involved a patient with a history of endometriosis who had previously undergone pelvic surgery.

Postpartum haemorrhage was also frequent, affecting eight women (61.5%), with a mean blood loss of 812.50 mL. Among these, five patients had an estimated blood loss superior to 1000 mL and required transfusion. The mean duration of hospital admission for the patients in this study was 4.85 days.

Regarding neonatal outcomes, there was one case of perinatal death (7.7%), and one admission to the neonatal intensive care unit (NICU), which occurred in a twin gestation (Supplemental Table S3). Notably, excluding the perinatal death, no newborn had an APGAR score below 5 at 5 min after birth.

## Discussion

The rate of uterine rupture in this series is slightly above that reported by other developed countries and in Europe.^18^ However, a retrospective study in Australia found a comparable prevalence.^8^ In this study, 11 out of 13 cases with uterine rupture (84.6%) occurred in women with a previous caesarean section, reinforcing the well-established association between uterine scarring and rupture risk. With the global rise in caesarean deliveries, and particularly the elevated rates observed in Portugal, it is anticipated that uterine ruptures during pregnancy may also increase. While this study does not explore the indications for caesarean deliveries, a more judicious approach to primary caesareans, especially in low-risk primiparous women, could help reduce long-term obstetric complications. Myomectomy is also recognized as a risk factor, but was not observed in this study.

The overall prevalence of rupture in induction after a previous caesarean section was high, as reported previously.^19^ At the hospital referenced in this study, oxytocin is the preferred method for inducing labour in such cases, while mechanical methods are rarely employed in patients with an unfavourable Bishop score. Cervical ripening with prostaglandins is not performed under any circumstances in this setting. During the study period, induction of labour in the low-risk population without maternal or fetal complications was preferentially performed at 41 weeks of gestation. This practice may have contributed to an even higher likelihood of uterine rupture.

Nowadays, trends have concentrated on reducing the likelihood of uterine rupture in patients with a history of caesarean section. Specialized techniques, such as ultrasound, can be used to assess scar thickness, though the results remain conflicting.^19^

It is well established that augmentation of labour with oxytocin is associated with an increased risk of uterine rupture, particularly in patients with a history of caesarean delivery. In this study, the rate of oxytocin augmentation was notably high (10/13). However, a review of clinical records revealed that the maximum infusion rates thresholds established by the service protocols were not exceeded in any group (144 mL/h for a maximum of 180 mL/h without caesarean scar; 84 mL/h for a maximum of 120 mL/h with previous caesarean), and no cases of tachysystole were observed in CTG. These findings underscore the critical importance of the cautious and judicious use of oxytocin during labour management.

Uterine rupture occurred in two cases without prior caesarean sections, emphasizing the need for vigilance even in pregnancies without typical risk factors. Other population-based studies frequently reported similar cases.^6,12,18–22^ The first case involved a primigravida with no notable medical or surgical history, who experienced uterine rupture following cervical ripening with misoprostol and labour augmentation using oxytocin. Cervical ripening was considered successful, with a favourable Bishop score (7) prior to the initiation of oxytocin. The second case featured a woman with spontaneous onset of labour. She had a history of infertility and pelvic surgery due to deep endometriosis, and a twin gestation achieved through in vitro fertilization, but no evidence of adenomyosis. Accordingly, a recent case-series^23^ highlighted the potential increased risk of uterine rupture during pregnancy among women who have had a prior resection of deep endometriosis.

Based on the literature, several other factors may play a role (probably less significant) in the likelihood of uterine rupture, including a short interval (<12 to <24 months) after a previous caesarean section – only one case in this series, abnormal fetal position, excessive amniotic fluid, abnormally invasive placentation, placental abruption, connective tissue disorders, adenomyosis, trauma and uterine abnormalities, all non-present.^4^

As described in previous studies,^24,25^ abnormal CTG was the most common sign of uterine rupture. In this study, a marked occurrence of prolonged deceleration and bradycardia was observed. Continuous monitoring of fetal heart rate during labour may play a crucial role in the early detection of uterine rupture. Loss of fetal station was observed in 2 of 13 cases (15.4%) in this series. Although not commonly reported in large studies, this sign has been described in the literature as a potential indicator of uterine rupture and may contribute to early recognition in selected cases.^26^ Patients also reported pain in the lower abdomen and shoulders, a symptom particularly relevant in women receiving epidural analgesia.

In this study, no maternal deaths were reported, and the overall perinatal mortality rate was 7.7%. This is slightly lower than rates reported in other studies, which vary between 13% and 26% depending on the population and context of uterine rupture.^18,24,25^ Uterine rupture resulted in longer hospitalizations than anticipated for caesarean section postpartum at the institution, typical 48 h. It was also associated with high rates of peripartum haemorrhage and need for transfusions, resulting in increased morbidity and costs.

The strength of this study lies in the comprehensive review of electronic hospital records for all patients, which was conducted to validate previously routinely coded data. This approach ensured consistency in the definition of complete uterine rupture and a high level of accuracy in patient outcome data.

### Limitations

This study has several limitations. Its retrospective design limits the ability to establish causal relationships, and the relatively small number of cases reduces the robustness of statistical analyses. These factors should be considered when interpreting the findings.

## Conclusion

The prevalence of uterine rupture in this study is comparable to or slightly higher than that reported in other developed countries. Uterine rupture, while rare, predominantly occurs during labour, although it is not confined to patients with a history of uterine surgery. Induction of labour and augmentation with oxytocin should be approached with caution to mitigate this obstetric emergency. Maintaining a high clinical index of suspicion is critical to prevent delays and facilitate prompt surgical intervention, thereby minimizing maternal and fetal morbidity and mortality.

## Supplemental Material

sj-docx-1-whe-10.1177_17455057251399891 – Supplemental material for Uterine rupture in pregnancy over 5 years: A retrospective descriptive study Supplemental material, sj-docx-1-whe-10.1177_17455057251399891 for Uterine rupture in pregnancy over 5 years: A retrospective descriptive study by António De Pinho, Francisco Martins Dos Santos, Olímpia Carmo, João Bernardes and Ana Reynolds in Women's Health

sj-docx-2-whe-10.1177_17455057251399891 – Supplemental material for Uterine rupture in pregnancy over 5 years: A retrospective descriptive study Supplemental material, sj-docx-2-whe-10.1177_17455057251399891 for Uterine rupture in pregnancy over 5 years: A retrospective descriptive study by António De Pinho, Francisco Martins Dos Santos, Olímpia Carmo, João Bernardes and Ana Reynolds in Women's Health
