# Supplementary material for: Uterine rupture in pregnancy over 5 years: A retrospective descriptive study
Source: Womens Health (Lond). 2025 Nov 21;21:17455057251399891. doi: 10.1177/17455057251399891 (PMC12640446; doi:10.1177/17455057251399891)
Supplement: sj-docx-1-whe-10.1177_17455057251399891 – Supplemental material for Uterine rupture in pregnancy over 5 years: A retrospective descriptive study [file sj-docx-1-whe-10.1177_17455057251399891.docx]

**STROBE Statement**

Uterine Rupture in Pregnancy over 5 Years: A Retrospective Descriptive Study

|  | Item Nº | Recommendation | Reported on page number |
| --- | --- | --- | --- |
| **Title and abstract** | 1 | (*a*) Indicate the study’s design with a commonly used term in the title or the abstract | Pag 1 |
|  |  | (*b*) Provide in the abstract an informative and balanced summary of what was done and what was found | Pag 2 |
| Introduction | | | |
| Background/rationale | 2 | Explain the scientific background and rationale for the investigation being reported | Pag 3 |
| Objectives | 3 | State specific objectives, including any prespecified hypotheses | Pag 3 |
| Methods | | | |
| Study design | 4 | Present key elements of study design early in the paper | Pag 4 |
| Setting | 5 | Describe the setting, locations, and relevant dates, including periods of recruitment, exposure, follow-up, and data collection | Pag 4 |
| Participants | 6 | (*a*) Give the eligibility criteria, and the sources and methods of selection of participants | Pag 4 |
| Variables | 7 | Clearly define all outcomes, exposures, predictors, potential confounders, and effect modifiers. Give diagnostic criteria, if applicable | Pag 4 |
| Data sources/ measurement | 8* | For each variable of interest, give sources of data and details of methods of assessment (measurement). Describe comparability of assessment methods if there is more than one group | Pag 4 |
| Bias | 9 | Describe any efforts to address potential sources of bias | Pag 4 |
| Study size | 10 | Explain how the study size was arrived at | Pag 4 |
| Quantitative variables | 11 | Explain how quantitative variables were handled in the analyses. If applicable, describe which groupings were chosen and why | Pag 4 |
| Statistical methods | 12 | (*a*) Describe all statistical methods, including those used to control for confounding | Pag 4 |
|  |  | (*b*) Describe any methods used to examine subgroups and interactions | Not applicable, since this study does not include subgroup analyses or interactions. |
|  |  | (*c*) Explain how missing data were addressed | Pag 4 |
|  |  | (*d*) If applicable, describe analytical methods taking account of sampling strategy | Pag 4 |
|  |  | (*e*) Describe any sensitivity analyses | Not applicable, this study is a descriptive study focusing on prevalence and clinical characteristics rather than statistical modeling. |
| Results | | | |
| Participants | 13* | (a) Report numbers of individuals at each stage of study—eg numbers potentially eligible, examined for eligibility, confirmed eligible, included in the study, completing follow-up, and analysed | Pag 5 |
|  |  | (b) Give reasons for non-participation at each stage | Not applicable. This study is a retrospective analysis of all confirmed cases of uterine rupture during the study period. There was no active recruitment or invitation for participation. |
|  |  | (c) Consider use of a flow diagram | Not applicable. All cases of uterine rupture recorded in hospital files were included in the study. Since there were no exclusions at different study stages, a flow diagram would not provide additional information. |
| Descriptive data | 14* | (a) Give characteristics of study participants (eg demographic, clinical, social) and information on exposures and potential confounders | Pag 5 |
|  |  | (b) Indicate number of participants with missing data for each variable of interest | Not applicable. There was no missing data. |
| Outcome data | 15* | Report numbers of outcome events or summary measures | Pag 5-6 |
| Main results | 16 | (*a*) Give unadjusted estimates and, if applicable, confounder-adjusted estimates and their precision (eg, 95% confidence interval). Make clear which confounders were adjusted for and why they were included | All estimates are unadjusted, as this study follows a purely descriptive approach. The data is presented in tables, reflecting the direct prevalence and clinical characteristics of the included cases without statistical adjustments. |
|  |  | (*b*) Report category boundaries when continuous variables were categorized | Not applicable. All continuous variables were analysed as means and standard deviations, without categorization. |
|  |  | (*c*) If relevant, consider translating estimates of relative risk into absolute risk for a meaningful time period | Not applicable. Given that this study is descriptive, relative risk was not calculated. |
| Other analyses | 17 | Report other analyses done—eg analyses of subgroups and interactions, and sensitivity analyses | Not applicable. The study does not involve sample subdivisions, interaction analyses, or sensitivity tests. The objective was to describe the prevalence and clinical findings of the included cases. |
| Discussion | | | |
| Key results | 18 | Summarise key results with reference to study objectives | Pag 9 |
| Limitations | 19 | Discuss limitations of the study, taking into account sources of potential bias or imprecision. Discuss both direction and magnitude of any potential bias | Pag 8 |
| Interpretation | 20 | Give a cautious overall interpretation of results considering objectives, limitations, multiplicity of analyses, results from similar studies, and other relevant evidence | Pag 7-8 |
| Generalisability | 21 | Discuss the generalisability (external validity) of the study results | Pag 7-8 |
| Other information | | | |
| Funding | 22 | Give the source of funding and the role of the funders for the present study and, if applicable, for the original study on which the present article is based | Not applicable, there was no funding for the conduction of this study. |

*Give information separately for exposed and unexposed groups.
