# Supplementary material for: Uterine rupture in pregnancy over 5 years: A retrospective descriptive study
Source: Womens Health (Lond). 2025 Nov 21;21:17455057251399891. doi: 10.1177/17455057251399891 (PMC12640446; doi:10.1177/17455057251399891)
Supplement: sj-docx-2-whe-10.1177_17455057251399891 – Supplemental material for Uterine rupture in pregnancy over 5 years: A retrospective descriptive study [file sj-docx-2-whe-10.1177_17455057251399891.docx]

**SUPLEMENTARY MATERIAL**

Table 1 - Baseline and obstetric characteristics in cases of uterine rupture.

| Maternal characteristics | All cases *n*=13 |
| --- | --- |
|  |  |
| Maternal age in years, mean (SD) | 33.85 (5.08) |
| BMI kg/m^2^, mean (SD) | 27.77 (3.77) |
| Scarred Uterus, n (%) | |
| Yes | 11 (84.6) |
| No | 2 (15.4) |
| Parity, n (%) | |
| 0 | 2 (15.4) |
| 1 | 9 (69.2) |
| ≥ 2 | 2 (15.4) |
| Gestational age in weeks, mean (SD) | 38.46 (1.81) |
| Previous caesarean section, n (%) | |
| 0 | 2 (15.4) |
| 1 | 10 (76.9) |
| ≥ 2 | 1 (7.7) |
| Interpregnancy interval after caeserean in months, mean (SD) | 94.64 (62.54) |
| Labour, n (%) | |
| Spontaneous labour | 10 (76.9) |
| Induction of labour | 3 (23.1) |

BMI, body mass index; SD, standard deviation.

Table 2 - Signs and symptoms of uterine rupture.

| Signs and symptoms | All cases *n*=13  n (%) |
| --- | --- |
| Abnormal CTG | 10 (76.9) |
| Abdominal pain | 3 (23.1) |
| Loss of fetal station | 2 (15.4) |
| Vaginal bleeding | 1 (7.7) |
| Shoulder pain | 1 (7.7) |
| Asymptomatic | 2 (15.4) |

CTG, cardiotocography.

Table 3 - Maternal and neonatal complications.

| Complications | All cases *n*=13 |
| --- | --- |
| Maternal complications n (%) | |
| Maternal death | 0 (0.0) |
| Postpartum haemorrhage | 8 (61.5) |
| Postpartum haemorrhage in mL, mean (SD) | 812.50 (258.78) |
| 500–1000 mL | 3 (23.1) |
| ≥1000 mL | 5 (38.5) |
| Blood transfusion | 5 (38.5) |
| Hospital admission in days, mean (SD) | 4.85 (2.79) |
| Hospital admission > 5 days | 5 (38.5) |
| Cervical injury | 1 (7.7) |
| Urinary tract injury | 1 (7.7) |
| Large-volume hemoperitoneum | 2 (15.4) |
| Chorioamnionitis | 3 (23.1) |
| Uterine haemostatic sutures | 1 (7.7) |
| Neonatal complications n (%) | |
| Perinatal death | 1 (7.7) |
| *Remaining Newborn n (%)* | |
| Fetal extrusion out of uterus | 9 (69.2) |
| NICU admission | 1 (7.7) - twin gestation |
| 5 min APGAR < 5 | 0 (0.0) |

APGAR, appearance, pulse, grimace, activity, and respiration; NICU, neonatal intensive care unit; SD, standard deviation.
